# Amine-Reactive Augmentation of Silk Fibroin Mats for Increasing Cargo Retention Capabilities

**DOI:** 10.3390/jfb17040161

**Published:** 2026-03-31

**Authors:** Kamali L. Charles, Yunhui Xing, Ellen L. Otto, Xi Ren, Phil G. Campbell, David A. Vorp, Justin S. Weinbaum

**Affiliations:** 1 Department of Bioengineering, Swanson School of Engineering, University of Pittsburgh, 4200 Fifth Avenue, Pittsburgh, PA 15260, USA; 2 Department of Surgery, School of Medicine, University of Pittsburgh, UPMC Presbyterian Hospital, F1281 200 Lothrop Street, Pittsburgh, PA 15213, USA; 3 Department of Biomedical Engineering, College of Engineering, Carnegie Mellon University, 5000 Forbes Avenue, Pittsburgh, PA 15213, USA; 4 Department of Mechanical Engineering, College of Engineering, Carnegie Mellon University, 5000 Forbes Avenue, Pittsburgh, PA 15213, USA; 5 Department of Mechanical and Materials Engineering, Swanson School of Engineering, University of Pittsburgh, 4200 Fifth Avenue, Pittsburgh, PA 15260, USA; 6 Department of Chemical and Petroleum Engineering, Swanson School of Engineering, University of Pittsburgh, 4200 Fifth Avenue, Pittsburgh, PA 15260, USA; 7 Center for Vascular Remodeling and Regeneration, University of Pittsburgh, 4200 Fifth Avenue, Pittsburgh, PA 15260, USA; 8 Department of Cardiothoracic Surgery, School of Medicine, University of Pittsburgh, 4401 Penn Avenue, Central Plant Building, Suite 3200, Pittsburgh, PA 15224, USA; 9 Clinical and Translational Sciences Institute, School of Medicine, University of Pittsburgh, Forbes Tower, Meyran Avenue Suite 7057, Pittsburgh, PA 15213, USA; 10 McGowan Institute for Regenerative Medicine, School of Medicine, University of Pittsburgh, 450 Technology Drive, Pittsburgh, PA 15219, USA; 11 Magee-Womens Research Institute, School of Medicine, University of Pittsburgh, 204 Craft Avenuee, Pittsburgh, PA 15213, USA; 12 Department of Pathology, School of Medicine, University of Pittsburgh, S-417 BST 200 Lothrop Street, Pittsburgh, PA 15261, USA

**Keywords:** silk fibroin, click chemistry, electrospinning, DBCO, conjugation

## Abstract

Silk fibroin (SF) is an ideal biomaterial for next-generation clinical wound dressings due to its biocompatibility and tunable mechanical properties. Cell therapies for wound healing have explored using SF as the base for delivering beneficial cargo; however, retention is poor due to exudate “wash out.” To address concerns with the premature release of cargo from SF-fabricated wound dressings, we utilized amine-reactive chemistry to conjugate SF mats with azido-reactive dibenzocyclooctyne (DBCO) that can then attach complementary azido-tagged cargo through chemoselective immobilization. SF mats were made using electrospinning of a 1:1 SF/PCL solution and were then conjugated with N-Hydroxysuccinimide-dibenzocyclooctyne ester (DBCO). PBS soaking was used for control SF mats. SF mats were then imaged and characterized using the following metrics: pore size, fiber alignment, fiber distribution, fiber diameter, ultimate tensile strength, tangent modulus, proteolytic degradation, absorption, and retention. Successful DBCO conjugation of SF mats was confirmed through the presence of the Az-Cy5 dye while exhibiting no significant changes to the DBCO SF mats in any of the tested metrics compared to controls. Our results provide evidence that the amine chemistry responsible for the DBCO conjugation does not alter important SF mat properties. This confirms that DBCO augmentation paired with Az-Cy5 tags may be a viable approach for immobilizing different therapeutic cargoes to aid wound healing efforts.

## 1. Introduction

Chronic wounds, described as open wounds persisting for over 3 weeks without notable signs of closure, are estimated to be present in ~3% of the United States population [1]. Common comorbidities that increase the prevalence of chronic wounds include diabetes and obesity, which are associated with 10.5% and 42.4% of wounds, respectively [1]. These conditions delay wound closure due to their association with peripheral arterial disease, which damages the vascular system and results in continual tissue necrosis without resolution. As the prevalence of these risk factors continues to increase annually, the financial burden of wound management for patients also rises. United States Medicare estimates that the annual cost of chronic wound care ranges from 28.1 to 96.8 billion dollars. This metric includes costs related to infection management and surgical interventions required for wounds with delayed closure [1], as infection prevalence increases with the duration that the wound remains open. Prolonged wound exposure to the external environment also increases the risk of bacterial contamination and infection, which can lead to limb amputation and increased mortality over time [1].

While acute wounds initially enter an inflammatory stage to remove debris through macrophage and protease activity, an overreactive inflammatory response can create conditions that complicate wound healing. Reactive oxygen species generated from protease and macrophage activity result in the secretion of pro-apoptotic bodies and both the activation and recruitment of additional pro-inflammatory cells [2,3,4]. Increases in inflammation resulting from tissue and extracellular matrix degradation, combined with patient vascular dysfunction, prevent the wound from transitioning to the proliferative phase of healing [2,3]. This disruption also prevents resident dermal cells from proliferating and migrating, thereby maintaining wound depth and exposure to the external environment. A major hurdle when designing biocompatible wound dressings is protecting the open wound from the microenvironment while also providing regenerative capabilities. Clinically, chronic wounds develop from common injuries such as diabetic foot ulcers, acute dermal trauma, and neuropathic pressure ulcers [5,6,7,8,9].

The development of a comprehensive wound dressing that incorporates materials suited to the wound environment [10,11] and that contains pro-regenerative [12] properties has the potential to reduce both the cost and frequency of interventions for patients undergoing chronic wound treatment [13,14,15,,16]. Silk fibroin (SF) is an ideal biomaterial for next-generation clinical wound dressings due to its biocompatibility, tunable mechanical properties, and potential to act as a scaffold for regenerative cargo [15,17,18,19]. However, SF does not inherently promote cellular activity such as proliferation or migration.

Two cytokines that could be incorporated into wound healing dressings are vascular endothelial growth factor (VEGF), which stimulates neo-vessel formation [20], and transforming growth factor-beta (TGF-β) [21], which promotes re-epithelialization through keratinocyte migration [8,21,22,23,24,25]. Regenerative therapies have sought to deliver cell or cell-based cargo containing these two cytokines for chronic wound treatment. However, retention of regenerative cargo at the wound site is poor due to exudate “wash out.” This limitation necessitates repeat dosing to achieve the desired therapeutic effect, which can result in off-target accumulation of the therapeutic agent in non-target organs [26,27,28,29,30,31,32]. To address concerns with delivering exogenous VEGF and TGF-β, here, we utilize amine-reactive chemistry to conjugate our SF mats with azido-reactive dibenzocyclooctyne (DBCO). This modification enables the SF mat to protect and display complementary azido-tagged regenerative cargo through chemoselective immobilization.

Chemoselective immobilization, or “click binding chemistry,” has emerged as a novel technique for biomaterial functionalization and has demonstrated its ability to immobilize desired biomolecules in collagen with superior efficacy and preserved bioactivity [27,33]. Given the high reactivity between click-reactive ligands and their biological and chemical inertness in natural biological systems, click chemistry could be a promising approach for improving retention of regenerative cargo at the target site. Furthermore, the alkyne- and azido-based functional groups required for the spontaneous cycloaddition are not typically found in biological systems, minimizing the potential for unintended reactions with organic molecules, thus providing a high specificity of binding [27,33,34]. By chemically attaching cargo to an immobile SF biomaterial reservoir, rapid clearance and off-target accumulation of the regenerative cargo would be reduced, enabling more efficient and targeted localized delivery. The goal of this study was to fabricate and chemically modify SF mats without altering their mechanical and functional properties in preparation for chemical decoration of regenerative cargo.

## 2. Methods

### 2.1. Silk Fibroin Isolation

Silk fibroin was isolated from mulberry *Bombyx mori* silk cocoons (Minkissy, item# 738661843208, Shenzhen, China) using previously described protocols [35]. Briefly, 5.0 g of silk cocoons were cut into several pieces, degummed in 0.02 M Na_2_CO_3_, and dissolved in 9.3 M LiBr (3213225, Sigma-Aldrich, 6000 N Teutonia Ave, Milwaukee, WI 53209, USA). Dissolved silk was then dialyzed using 3.5 kDa cassettes (Cat #: 66110) for 24 h against MilliQ water (18.2 Ω). The resulting regenerated silk protein was then purified using centrifugation and decantation, which was performed twice. Purified regenerated silk protein was then pipetted into molds and allowed to freeze for ≥4 h before being lyophilized overnight, creating SF pellets. Lyophilized SF pellets were then removed from molds and stored at 4 °C in a sealed container until used for formulating electrospinning solution, as detailed in Section 2.2.

### 2.2. Silk Mat Fabrication

**Figure 1:**
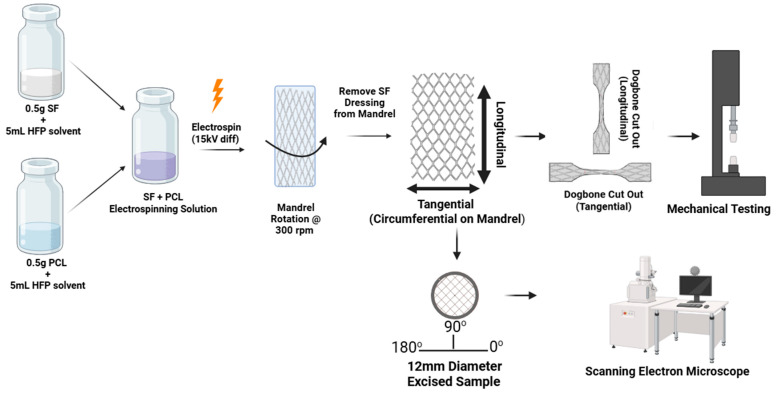
Schematic showing fabrication of SF mats. SF and PCL were first separately dissolved in HFP, then mixed to create a homogeneous solution used to electrospin fibers targeted at a rotating 25 mm diameter mandrel. The resultant SF mat was then removed and sectioned into smaller specimens for mechanical testing, morphological analysis via SEM, and absorption and retention testing. For mechanical testing, longitudinally and tangentially oriented specimens were cut from the mats, as shown. For morphological, absorption, and retention testing, separate circular samples were excised from the mat as shown and tested, as described below.

SF mats were fabricated by electrospinning SF, as shown in Figure 1. For this, 0.5 g of lyophilized SF was dissolved in 5 mL of 1,1,1,3,3,3-Hexafluoro-2-propanol (105228, HFP, Sigma Aldrich). Separately, 0.5 g of polycaprolactone (440744, average MW 80,000, Sigma-Aldrich) was dissolved in 5 mL HFP. Both dissolved solutions were then combined and mixed until a homogeneous electrospinning solution was formed. The following parameters were then used on an IME electrospinning machine (IME Technologies, 5581 WG Waalre, The Netherlands) to fabricate the SF mats: flow rate (100 µL/min), translational injector speed (50 mm/s), tip to mandrel distance (15 cm), rotational mandrel speed (300 rpm), mandrel diameter (25 mm), applied voltage difference (15 kV), temperature (~25 °C), and humidity (~47%). A 150 msec turn delay was incorporated into the fabrication procedure after thickness measurements across the length of the SF mat presented in a bell-shaped fashion, with higher thickness towards the inner third and thinning in the outer third portion. This modification to our protocol improved end thickness of SF mats for uniformity in thickness across the length of our material, which was confirmed using SEM imaging at 2000× magnification, providing more usable material for SF mat characterization and functional testing. A total of 3.1 mL of electrospinning solution was used. The electrospun product was removed from the mandrel following a longitudinal cut, creating a rectangular SF mat, which was then stored at room temperature (RT ~20 °C) in a sealed plastic bag.

### 2.3. DBCO Conjugation of SF Mats

SF mats were sprayed with 70% ethanol and left to dry at RT for 10 min. Mats were then washed three times using phosphate-buffered saline (PBS, pH: 7.4, cat # 10010023, 5791 Van Allen Way, Carlsbad, CA 92008, USA, Thermo Fisher Scientific) to remove excess ethanol. Two 50 mL vials of 10% DMSO solutions in phosphate-buffered saline were prepared, and NHS-DBCO (Click Chemistry Tools, catalog #A133, 8341 E. Gelding Drive, Scottsdale, AZ 85260, USA) was added to a single vial to generate a final concentration of 200 µM. The mats were then transferred to containers and submerged in either 200 µM N-Hydroxysuccinimide-dibenzocyclooctyne ester (DBCO) solution or 10% DMSO vehicle control (CNTL) solution for 3 h at RT with agitation. SF mats were then intensively washed (3 × 3 min followed by 3 × 30 min) in 10% DMSO in phosphate-buffered saline to remove excess DBCO solution, as well as any residual HFP that had not dissolved during the electrospinning process. SF mats were allowed to dry at RT under a fume hood overnight and then stored in a sealed plastic bag at RT.

### 2.4. DBCO Conjugation Confirmation

For this paper, the only conjugate azido agent used to bind the DBCO-labeled SF mats was Az-Cy5 dye (Click Chemistry Tools, catalog #AZ118, 8341 E. Gelding Drive, Scottsdale, AZ 85260, USA) as a proof of labeling confirmation. Az-Cy5 was added to a final concentration of 15–20 µM in 50 mL PBS. Multiple circular sections were excised from the DBCO and CNTL SF mats. Excised sections were then submerged in the Az-Cy5 solution for 1 h at RT with agitation. SF mats were then intensively washed, and gross inspection showed retention of conjugate dye. Additionally, fluorescent microscopy was performed using a Cy5 filter to capture and compare the fluorescent signal produced by both DBCO SF and CNTL SF.

### 2.5. SEM and Morphological Assessment

Excised 4 mm diameter sections of both DBCO and CNTL SF mats were taken while also demarking the longitudinal directionality in relation to the SF mat. Samples were then sputter-coated and imaged under a scanning electron microscope (JEOL JSM-7800 FE SEM, 11 Dearborn Road, Peabody, MA 01960, USA). SEM images were collected from three separate samples of both groups of mats at a magnification of 2000× to be used for subsequent comparative analysis. A magnification of 2000× was chosen, as it gave the best overall representation of the full SF mat surface while also including enough SF fibers for additional fiber analysis.

Generated segmented images were further processed using our previously published MATLAB ver.R2023a-based method for morphological analysis, including pore size, porosity, fiber diameter, fiber angular distribution, and tortuosity [36].

Collected SEM images were uploaded into ImageJ software (1.4x) and converted to 8-bit images for further processing. Images then underwent thresholding to create black and white images to be used for visualizing and quantitative analysis. These black and white images were then further processed using the “measure” feature to calculate average pore size for both DBCO and CNTL SF mats. Further quantitative analysis required the previously generated black and white images to be fitted with a fiber skeleton, allowing fiber thickness, fiber length, and end-to-end fiber distance to be calculated. While fiber diameter can be measured in microns and fiber angular distribution in degrees, we note that tortuosity is considered a dimensionless or unitless metric as it is defined as the ratio of the total measured path length of a fiber from one end to another divided by the straight-line (shortest) distance between the two ends of that same fiber (thus, a tortuosity of 1.0 is a completely straight fiber). Using a secondary MATLAB code, fiber angle and distribution were calculated using an orthogonally placed *x*- and *y*-axis based on the orientation of the image. Images were processed using a 2D Fourier-transform method to create circular graphs, plotting fiber angles and relative distribution of fibers at each angle. Porosities of the SF mats were calculated for a randomly defined region of interest for each sample, which was then broken into a 100 × 100-pixel frame to generate a ratio of white pixels to the total pixel count within the cropped image. Porosity was then calculated by assessing the ratio of black pixels to the total pixel count and expressing it as a percentage [36].

### 2.6. Mechanical Testing

Dog-bone-shaped specimens (length 2.2 cm × width 0.4 cm) were excised from SF mats, both oriented along the longitudinal direction of the SF mats and tangential or perpendicular to that (i.e., circumferential direction when oriented with the cylindrical electrospinning target). Samples were taken from both longitudinal and tangential directions to assess and compare intrinsic mechanical properties of the SF mats in both directions. While we understood SF is a viscoelastic material and would normally require preloading, we did not perform this procedure, as it was expected that if all samples were either preloaded or not, then creep and stress relaxation would equally be altered or unaltered and have a negligible impact on the load-to-failure testing. Uniaxial tensile strength testing was performed on both CNTL and DBCO SF mats using a tensile testing device (Instron, 825 University Ave, Norwood, MA 02062, USA, model 5543A, load cell capacity = 111.2 N) with *n* = 12 for each direction. The wide ends of the dog bone samples were reinforced with a layer of masking tape followed by an outer layer of duct tape prior to loading into pneumatic grips on the Instron device to ensure uniform load distribution and mechanical failure in the thin central region of the sample. During uniaxial tensile testing, samples were placed under tension at a constant elongation rate of 3 mm/min until failure occurring in the center of the dog-bone-shaped structure was observed. The thickness of the sample was calculated using 3 SEM images per sampled SF mat prior to tensile testing. Applied forces on both longitudinal and tangential samples were recorded during testing through ultimate failure, ranging from 0.94 N to 2.99 N and 0.60 N to 1.86 N, respectively. From the mechanical testing, stress–strain curves were generated and, using measured length, width, and thickness, were processed to calculate both ultimate tensile strength (UTS) and tangent modulus (Mod). These two metrics were then graphed and compared between conjugation groups and sample orientations.

### 2.7. Silk Degradation

Enzymatic degradation profiles of 12 mm DBCO and CNTL SF mats were determined using Protease XIV and vehicle control using PBS. After fabrication of SF mats, the initial weight was recorded. Samples of dry SF mats were placed into a Petri dish and incubated at 37 °C with a 1 mL enzyme solution (2 U/mL in PBS) containing activated Protease XIV (P5147, Sigma-Aldrich). Following an initial 24 h, and every subsequent 48 h, the mat sample was removed from the solution, dried overnight under a fume hood, and weighed. Measurements were repeated for 7 days, and the remaining mass percentage was calculated for each day using Equation (1).

(1) % mass remaining = (W_t_/W_1_) × 100

Here, W_t_ is the dry weight of the sample at time t, and W_1_ is the initial dry weight of sample prior to treatment. After each measurement, fresh protease solution was reapplied to each SF sample, and the protocol timeline continued.

### 2.8. Exudate Functional Testing

Simulated exudate absorption/retention testing was performed by excising silk samples from either DBCO or CNTL SF mats using a 12 mm wound biopsy punch. Initial weights were recorded for each sample prior to incubating each individual sample in PBS for 30 min at RT. Following incubation, each sample was weighed at two additional timepoints: immediately after removal from PBS solution and post-30 s compression using a 1 kg weight. For calculating absorption and retention, SF mat thickness measurements were calculated by averaging cross-sectional height measurements from SEM images of randomly selected SF mats from three separate lots. All sample weights were used to calculate absorption and retention of all samples, as described in Equations (2) and (3), with comparisons made between DBCO and CNTL SF mats.

(2) Absorption (g/mm^3^) = (W_2_ − W_1_)/(πr^2^ × T)

(3) Retention (g/mm^3^) = (W_3_ − W_1_)/(πr^2^ × T)

Here, W_1_ is the initial dry weight of a sample, W_2_ is the sample weight immediately removed from PBS solution following 30 min submersion in PBS, W_3_ is the sample weight after 30 s of compression, and T is the sample thickness. Thickness was measured for all SF mats, which were found to be no different (*p* = 0.7695) between DBCO and CNTL, and we used the average of 65 μm for T. Radius (r) was 6 mm, as all samples were collected using a 12 mm diameter wound biopsy punch.

### 2.9. Statistics

Sample size analysis was conducted a priori using G-Power software (version 3.1, Heinrich-Heine-Universtät Düsseldorf), with α = 0.05 and power = 0.80 to calculate size needed per experiment group. Statistical tests used in our analyses were multiple paired *t*-tests and ordinary one-way ANOVA with multiple comparisons between experimental groups.

## 3. Results

### 3.1. DBCO Conjugation Was Confirmed Using Az-Cy5 Dye

**Figure 2:**
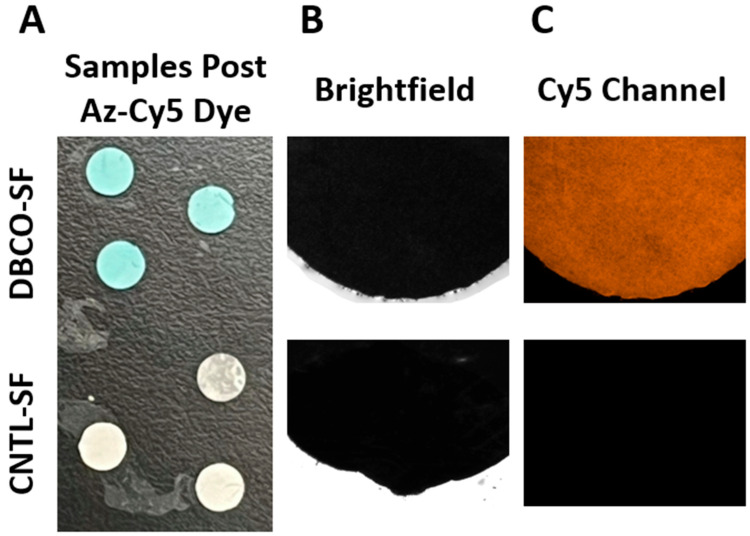
Confirming successful functional DBCO conjugation to SF mats. (**A**) Photograph of 4 mm diameter circular samples of SF mats. The three samples at the top show DBCO-conjugated mats that have retained the blue Az-Cy5 dye, while the bottom three samples show the CNTL without the blue staining following the extensive wash protocol. (**B**) Images collected by inverted microscope showing 4× magnification images of representative SF mat samples following Az-Cy5 staining and washing. The top image shows a representative sample from the DBCO group, and the bottom image is a representative sample from the CNTL group, both imaged under brightfield lighting. (**C**) The same representative samples from each group were imaged through the Cy5 fluorescent channel. Only the DBCO-conjugated SF mats at the top display fluorescence, while the CNTL sample on the bottom is not visible.

After incubation with Az-Cy5 dye and following intensive washes, the DBCO SF mats showed a strong blue color (Figure 2A) that was not seen in CNTL SF. Both DBCO SF sections and CNTL SF sections were imaged under a bright field (Figure 2B) to identify regions of interest. Fluorescent imaging was then performed through the Cy5 channel (Figure 2C), demonstrating clear fluorescence in sections incubated with DBCO, while CNTL SF demonstrated no fluorescence.

### 3.2. Morphological Assessment Demonstrated Consistency Between DBCO and CNTL

**Figure 3:**
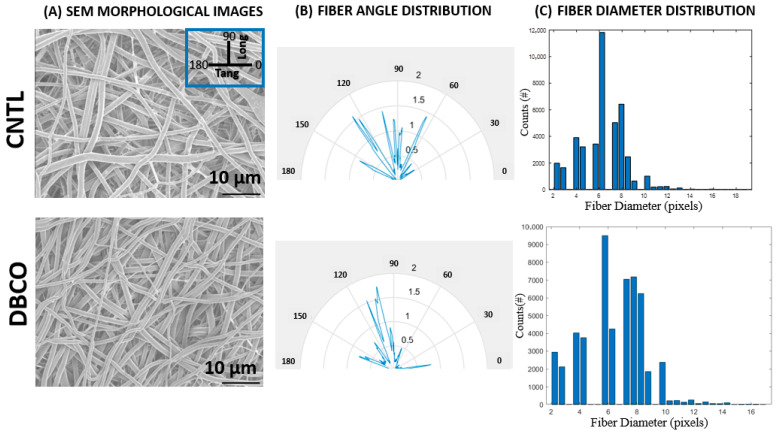
Consistent electrospun SF mat morphological characteristics. (**A**) Raw SEM images taken at 2000× magnification showing gross SF fiber morphology from both DBCO- and CNTL-conjugated SF mats. (**B**) Graphical representation of the distribution of fibers for both DBCO and CNTL SF mat samples from 0° to 180°, with values between 0° and 30° and 150° and 180° being closest to a tangential orientation, and those between 60° and 120° being closest to a longitudinal orientation. The histogram peaks were normalized across samples from zero to two. (**C**) Bar graphs showing the diameter distribution of SF fibers. No appreciable differences were noted between groups in any of these assessments.

SEM images of a representative sample from both DBCO and CNTL SF mats are shown in Figure 3A. Two-dimensional porosity measurements revealed 65.5% ± 0.61% for CNTL and 64.2% ± 3.59% for DBCO SF mats. Samples demonstrated consistent fiber distribution across both mat types, with large portions of fibers oriented so as to favor the longitudinal direction (90°, recall Figure 1) of the SF mat between angles of 60 and 120°. In contrast, fibers along the tangential direction were oriented between the 30° intervals of 0–30° and 150–180° (Figure 3B). Tortuosity measurements showed an average of 1.0 ± 1.9 × 10^−3^ for CNTL- and 1.0 ± 4.8 × 10^−3^ for DBCO-conjugated SF mats. That is, there was no appreciable tortuosity to the SF fibers in either the CNTL or DBCO SF mats. Mean fiber diameters for both DBCO and CNTL-conjugated SF mats were calculated at 6 pixels ± 2 pixels for both DBCO and CNTL groups (Figure 3C), which, when converted using the calculated 213 pixels (10 μm for 2000× magnification), equated to 0.28 μm ± 0.09 μm.

### 3.3. Mechanical Properties Were Unaltered Between Treated Groups

**Figure 4:**
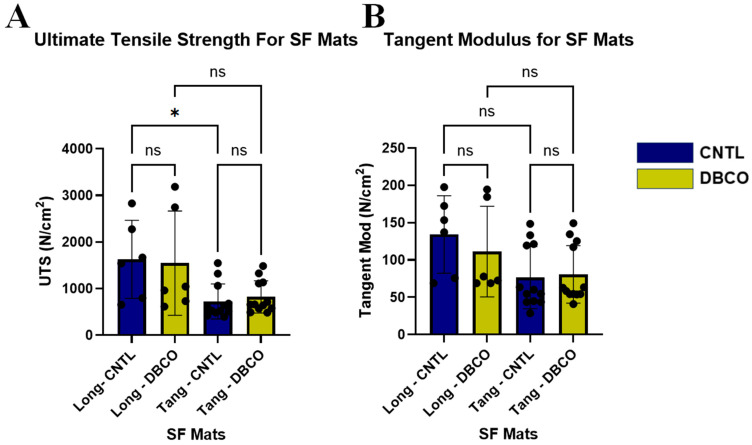
Retention of mechanical strength following conjugation. (**A**) Comparison of UTS values for longitudinal and tangential samples taken from DBCO and CNTL SF mats. (**B**) Comparison of tangent modulus between longitudinal and tangential samples taken from DBCO and CNTL SF mats. * *p* < 0.05 (exactly at 0.03). ns: no significance.

Uniaxial tensile testing on longitudinally sampled sections of both DBCO SF and CNTL SF, *n* = 8 per group, attained peak forces ranging from 0.94 N to 2.99 N for longitudinal sections and 0.60 N to 1.86 N for tangential samples. Analysis of the stress–strain data and extracted tensile strength and stiffness showed no significant difference between DBCO and CNTL groups in the same direction; however, a significant difference was seen between directions for the CNTL group (*p* < 0.05) but not the DBCO group (Figure 4A). Repeated testing of samples collected from both longitudinal and tangential directions for analysis of Mod in the SF mats reported no significant difference between any of the experimental groups (Figure 4B).

### 3.4. Pore Size and Degradation Assessment Shows No Difference Between DBCO and CNTL

**Figure 5:**
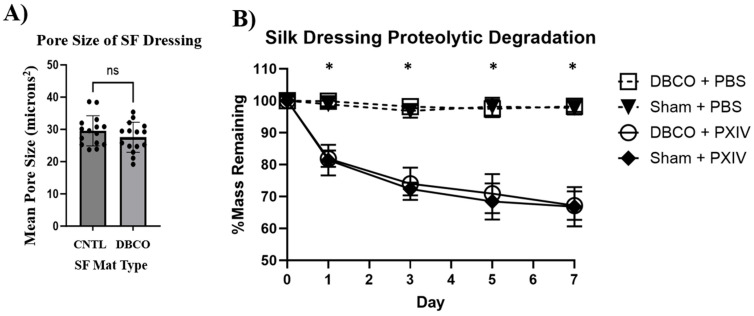
Consistent pore size and degradative profile of SF mats post-conjugation. (**A**) Bar graph comparing mean ± SEM pore size in CNTL- and DBCO-conjugated SF mat samples (*n* = 15 per group). No significant differences between groups were observed. (**B**) Degradation of DBCO and CNTL SF mat samples under treatment with either PBS (control) or Protease XIV over 7 days. The *Y*-axis shows the percentage of mass remaining of the measured sample compared to its initial (Day 0) starting weight. Comparisons were made between all four experimental groups, showing that the presence of DBCO had no effect (*p* = ns) on the degradation profile of SF mats and that PXIV significantly (*p* < 0.001) and similarly degraded both DBCO and CNTRL SF mats. * *p* < 0.001. ns: no significance.

Two-dimensional images of SF mats demonstrated consistent pore sizes with calculated means of 27.6 µm^2^ ± 4.64 µm^2^ and 29.6 µm^2^ ± 4.66 µm^2^ for DBCO and CNTL SF mats, respectively (Figure 5A), prior to starting degradation testing. Over seven experimental test days, SF mat samples left in PBS alone saw no significant decrease in mass between DBCO and CNTL SF mats compared to their initial dry weights (Figure 5B). Samples treated with Protease XIV demonstrated a ~18% reduction in initial mass within 1 day, then a steady decline over the remaining 6 days. Both DBCO and CNTL samples experienced the same rate of mass reduction, displaying no significant difference between sample types. Significant differences were only displayed between comparisons of treatment conditions.

### 3.5. Absorption and Retention Assessment Demonstrates Consistent Functionality Among Conjugation Types

**Figure 6:**
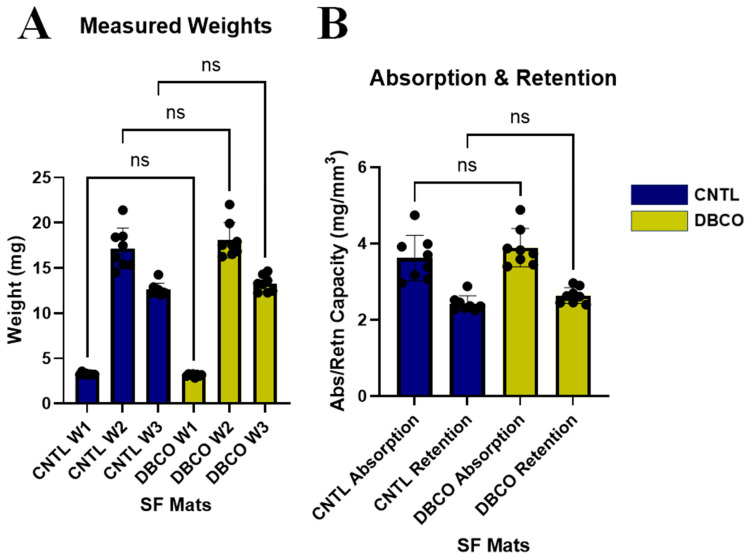
Consistent SF mat absorption and retention capability. (**A**) Measured weights of the SF mats at each of the three hydration conditions (W1 = initial dry weight; W2 = weight following saturation with PBS; W3 = weight following 1 kg compression over 30 s). No significant differences were found in any of the weights between DBCO and CNTL SF mats. (**B**) Absorption and retention values also showed no significant differences between CNTL and DBCO groups.

Initial dry weights (W_1_) of the CNTL and DBCO SF mats were 3.28 mg ± 0.13 mg and 3.13 mg ± 0.15 mg, respectively (Figure 6A). Following 30 min submersion in PBS, both groups of SF mats demonstrated an increase in weight (W_2_) of 17.17 mg ± 2.25 mg and 18.08 mg ± 1.96 mg for CNTL and DBCO, respectively (Figure 6A). All samples of both groups experienced a decrease in weight following 1 kg compression for 30 s (W_3_), being 12.59 mg ± 0.72 mg and 13.26 mg ± 0.91 mg for CNTRL and DBCO SF mats, respectively (Figure 6A). Comparisons between both CNTL and DBCO SF mats demonstrated no statistical difference in W_1_, W_2_, or W_3_.

Absorption and retention values calculated from Equations (2) and (3), respectively, were 3.62 g/mm^3^ ± 0.60 g/mm^3^ and 2.42 g/mm^3^ ± 0.21 g/mm^3^, respectively, for CNTL samples and 3.89 g/mm^3^ ± 0.50 g/mm^3^ and 2.63 g/mm^3^ ± 0.21 g/mm^3^, respectively, for NHS-DBCO absorption and retention were (Figure 6B). Similar to our findings when comparing weights at our three determined timepoints, absorption and retention values showed no statistical significance when comparing CNTL to DBCO SF mats.

## 4. Discussion

Morphological testing showed no significant differences between DBCO and CNTL in fiber characteristics, indicating homogeneity in the electrospinning procedure. As with electrospinning any biomaterial, the sinusoidal shape of the injected SF solution, resulting from the shift from ohmic to convective electrical flow during electrospinning [37], initially resulted in SF mats with bell-shaped thickness gradients across the target mandrel. A correction to this event was needed to create a more uniform SF mat to increase consistency during in vitro testing. Implementation of the 150 msec turn delay increased the working area of the fabricated SF sheet by applying gradual increases of the SF solution at each horizontal injector turn to produce larger quantities of SF mats for in vitro testing. Fiber diameter among our fabricated SF mats showed a similar distribution to other silk-based, electrospun dressings [13,15,38], indicating that solution consistency, target distance, and voltage gap were appropriate for fabrication. Consistency between both groups in fiber diameter and pore size provides evidence that amine chemistry responsible for the DBCO conjugation did not disrupt fiber formation, nor did it sheathe silk fibers.

Characterization of the SF mats, including mechanical assessment, was performed to evaluate any potential damage to the fabricated SF mat due to DBCO conjugation. Uniaxial tensile testing of the SF mats sampled from both longitudinal and tangential directions was performed, assessing the UTS and the Mod in both planar directions of the SF mats. Previous studies on SF wound dressings or electrospun materials have reported variable mechanical metrics due to the incorporation of additional biomaterials alongside silk [13,15,17,18,38,39,40]. However, as wound dressings are not expected to experience tension or stress strong enough to cause material failure, most studies report this variability as acceptable for experimental usage. Significant differences were not found when comparing CNTL- to DBCO-conjugated SF mats within the same directional orientation; however, comparisons of longitudinal and tangential test samples were statistically different. When we compare longitudinal and tangential metrics, our SF mats demonstrate anisotropic characteristics with higher ultimate tensile strength and tangent modulus in the longitudinal direction (across the length of the mandrel). Consistency across all fabricated SF mats allowed for statistically similar metrics such as pore size and porosity.

While DBCO conjugation has not yet been established using SF biomaterials, it has been previously reported for collagen fibers [27,33]. In a separate study, the complementary azido tag was conjugated to SF extracted from cocoons of fifth-instar larvae of the H06 transgenic *B. mori* silk worms after feeding them an artificial diet containing AzPhe (0.05% in dry diet) [34]. The AzPhe building block was then utilized in protein synthesis in the posterior silk glands of the silkworm. As a result, the silk cocoon generated from the silkworm contained the azido tag distributed at approximately two azido residues per SF molecule [34,41]. However, based on the reaction between the amino acid sequences within the SF primary structure and the reactive DBCO, we can infer that our DBCO labeling is occurring primarily at the N-terminus and side chains of amine-containing R groups of all polypeptide chains within the SF mat.

SF mats are expected to exhibit an initial burst in degradation over the first 24 h, followed by gradual decay over the course of their wear time. This time frame is consistent within the literature when using both protease K and XIV on SF biomaterials [35,42,43]. We also note that we expect the degradation of our mat will gradually taper due to the 1:1 SF/PCL ratio, wherein the PCL portion lacks sensitivity to protease effects. In Figure 5B, we see that after 7 days of exposure to protease XIV, our SF samples start to plateau at the 70% mass remaining section, also indicating that 30% of the material mass was degraded. This is like what we would expect, as electrospun silk sheets have been shown to degrade by approximately 70% within 15 days [44,45,46]. Given that our SF mat construct has approximately 50% resistance to proteolytic degradation (recall Figure 5B), if we use the expected degradation ratio of 70% per day, we quantitatively expect 32.7% mass loss within 7 days, which is consistent with our findings. While anticipating the exact mass loss due to SF degradation that will occur in vivo is difficult, we do understand that macrophages in the wound bed will selectively degrade the silk component, thus releasing bound cargo while maintaining the PCL base for clinician and patient handling. In the proteolytic environment of chronic wounds, host macrophages, neutrophils, and reactive oxygen species are expected to cleave our SF mat, releasing the regenerative cargo. This cargo can then interact with the resident inflammatory macrophages to modulate their phenotype, as well as affect subsequent vascular cells and/or fibroblasts in the healing wound tissue [47,48,49,50,51]. The pore size and total porosity across the surface of the SF mats enable the penetration of proteolytic wound exudate, allowing the SF mat to degrade at a consistent rate regardless of chemical modification. During our absorption and retention testing, we also discovered that our SF electrospun mats reached full saturation during absorption testing, as there was no significant difference in absorption metrics between 1 h and 24 h. This was noted as we initially collected the data seen in Section 3.5, followed by taking *n* = 4 DBCO-conjugated electrospun mats and allowing each sample to remain submerged for 24 h in PBS at RT. W_1_ and W_2_ were collected, and the resultant absorbance mean was calculated at 3.1 mg/mm^3^ ± 0.3 when compared to 1 h metrics for absorbance, 3.9 mg/mm^3^ ± 0.5. We do, however, note that this sample size would need to be increased for better comparison while also testing a 72 h timepoint and submerging solutions that simulate exudate ion content better than PBS. For clinical translation, we anticipate the need for additional exudate management and secondary dressings for venous wound etiology or lymphatic dysfunction.

Quantitatively, our study is limited by the inability to accurately assess the total density of DBCO molecules that have been attached to the SF fibers. This also limits the ability to accurately determine how much azido-labeled cargo binds to our DBCO-SF mats versus how much adheres due to lodging within pores, ionic bonding, or aggregate adhesion on the biomaterial. One potential solution to acquire quantitative data related to DBCO seeding density is to utilize a radioiodination technique to label our therapeutic cargo of interest. Using this technique, one could assess the radioactive decay over time, which should be representative of the cargo release from both DBCO-conjugated and CNTL SF mats. However, our current results showed successful Az-Cy5 binding to DBCO-SF biomaterials, therefore demonstrating that the azido-alkyne cycloaddition produces large, target-specific binding locations for purposely modified cargo.

While electrospinning creates a randomized network of SF fibers, the target surface for our SF mat was a 25 mm diameter, cylindrical, rotating mandrel. The cylindrical shape of the mandrel, combined with the consistent horizontal translation of the injector, provided secondary organization to the ejected fibers. Sole usage of a rotating, cylindrical target for the injected SF solution is another limitation of this study. Testing a flat, stationary target or a flat, orbital moving target may have reduced the likelihood of producing anisotropic SF mats. In these cases, the stable variables during the electrospinning process would be related to the flow rate of the solution and the translational speed of the injector. Additionally, shear stress may influence the efficacy of the SF mat in vivo and clinically if the target wound bed has minimal to no exudate, as can occur with arterial insufficiency. This occurrence may cause increased friction of the SF mat between the wound bed and the secondary dressing placed superficially. However, clinical observation of a wound bed with little to no exudate would signal the addition of a hydrating fluid to keep deep tissues alive while also preventing the SF mat from adhering directly to the wound bed as a “wet to dry” dressing.

While this study only utilized Protease XIV for its degradation studies, we expect the exudate of a chronically inflamed wound to have variable proteolytic profiles due to multiple enzymes being present. In anticipation of this occurrence, the resultant in vitro experiments are primarily used as a baseline for the degradation of our SF mats. Repeat degradative testing will occur once we conduct future in vivo studies, where tissue will be excised from our chronic wound model and made into an inflammatory, proteolytic solution that can be used for in vitro degradative tests. Additionally, SF mats used during in vivo studies can be salvaged, dried, weighed, and imaged using SEM to compare degradative profiles between in vitro and in vivo studies directly, so long as the initial weights of dressings pre- and post-regenerative cargo loading have occurred.

The abundance of DBCO conjugation sites on our SF material creates an opportunity to assess chemoselective binding of azido-labeled cargo, thus testing a new method for the controlled release of specific therapeutic agents. We hypothesize that this novel method will improve upon current soak loading and impregnating techniques currently implemented, as many click-bound agents will remain attached to the SF fibers and only detach as proteolytic enzyme activity cleaves the amino acid chain in the silk. However, we also note that this technique does not dampen the ability for cargo to be impregnated or embedded. Azido-labeled and non-labeled cargo can be embedded concurrently along non-conjugated fibers and within pores of the fabricated biomaterial. The duality of utilizing click chemistry in turn provides a potential way to have and alter burst release of a product while also ensuring controlled, sustained release over a tunable period. The ability to specifically and non-specifically adhere cargo to the SF fibers and retain the same different therapeutic cargo lodged within pores throughout the material can potentially increase delivered payloads to the wound bed. As a primary dressing, we do not anticipate the direction-dependent difference in ultimate tensile strength or tangent modulus to play a role in the efficacy of our SF mat. We, however, believe that conjugating the SF mat will enhance its drug delivery capabilities without causing any negative alterations to our biomaterial.

The initial promise of our SF biomaterial augmentation lays the foundation for future studies, where we intend to utilize mesenchymal stem cell-derived extracellular vesicles as our bioactive therapeutic cargo to covalently bind to our DBCO-conjugated SF wound dressing. These extracellular vesicles contain the neatly packaged secretome of their parent cells and have gained increasing interest in regenerative medicine due to their retention of MSC-like bioactivity, without the drawbacks of MSCs themselves. With current delivery methods of extracellular vesicles to diseased tissues being local injection and topical administration, this cargo is prone to clearance in wound exudates requiring multiple dosages. This limitation provides an excellent opportunity to assess our novel chemoselective immobilization technique in an in vivo wound healing model using a well-studied regenerative cell-based therapeutic.

## 5. Conclusions

In this study, we successfully fabricated and conjugated silk fibroin electrospun mats with DBCO, enabling an additional method for loading SF mats with pro-healing cargo. Additionally, our group conducted a series of characterization experiments, including SEM morphological assessment, pore size analysis, proteolytic degradation, and exudate absorption/retention. Within all conducted tests, we were able to demonstrate that the process of DBCO conjugation on SF mats caused no significant alterations to the structure and function of our biomaterial. These findings help support further exploration of our novel approach to increase the cargo retention on SF biomaterials using a bioactive agent as our cargo of interest in future studies. These future studies are required to further investigate azido-labeling of other types of regenerative cargo and assess both binding affinity and cargo release from altered silk through in vitro and in vivo experiments. Overall, this study provides a foundation for future work to generate clinically relevant data for drug delivery using click chemistry as a binding method of cargo to SF biomaterial.

## Data Availability

The original contributions presented in the study are included in the article; further inquiries can be directed to the corresponding author.

## Abbreviations

The following abbreviations are used in this manuscript:

DBCO	N-Hydroxysuccinimide-Dibenzocyclooctyne
CNTL	Control
SF	Silk Fibroin
UTS	Ultimate Tensile Strength
Mod	Tangent Modulus
